# Supplementary material for: Deconvolution of single-cell multi-omics layers reveals regulatory heterogeneity
Source: Nat Commun. 2019 Jan 28;10:470. doi: 10.1038/s41467-018-08205-7 (PMC6349937; doi:10.1038/s41467-018-08205-7)
Supplement: Supplementary file 1 — Supplementary Information [file 41467_2018_8205_MOESM1_ESM.pdf]

Deconvolution of single-cell multi-omics layers reveals regulatory heterogeneity

Liu *et al*.

Supplementary information

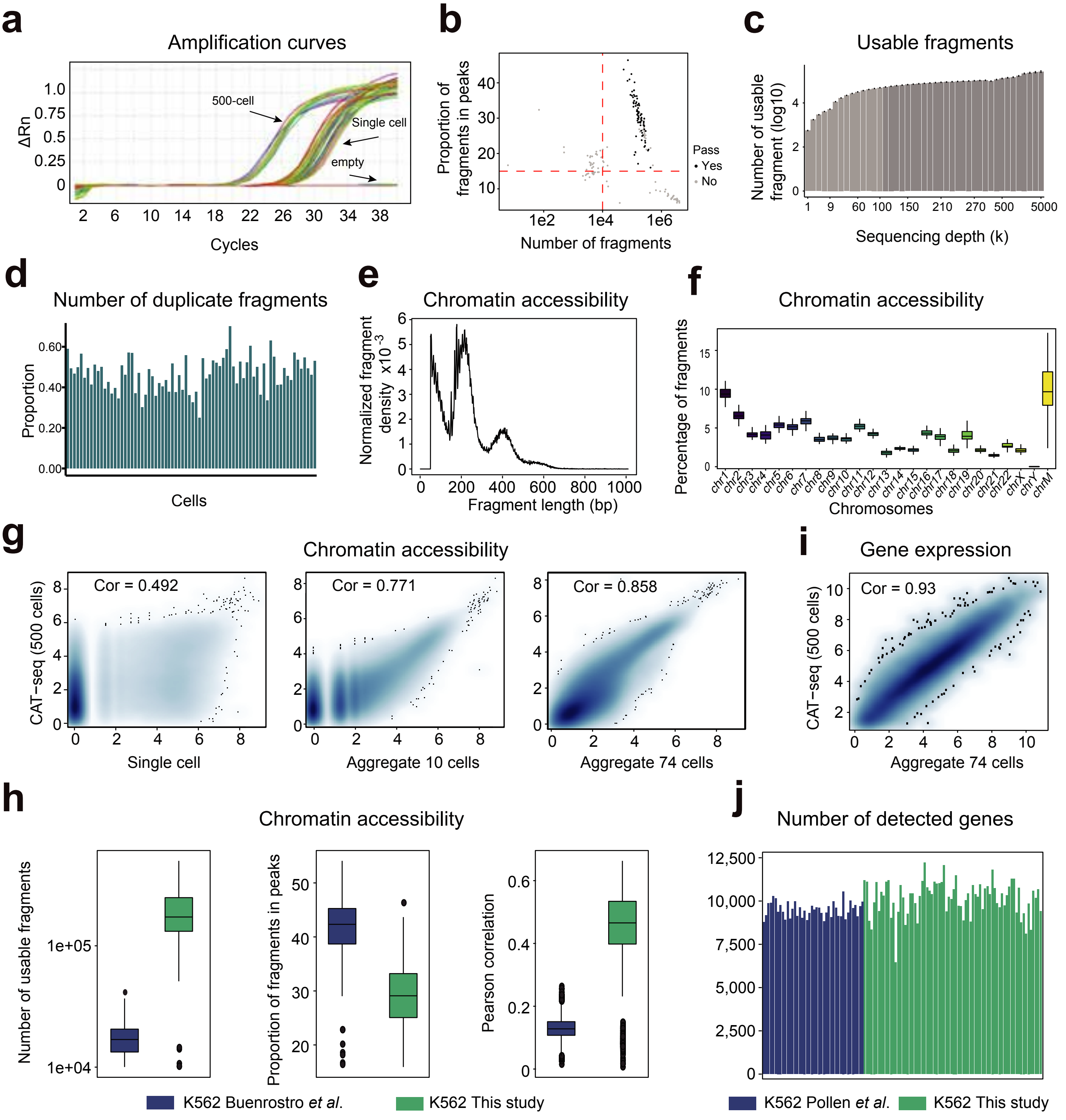

Supplementary Figure 1: Quality metrics of scCAT-seq data. (a) qPCR amplification curve using materials in the bottom of wells after the separation step of the scCAT-seq protocol. Wells containing 0, 1 and 500 cells were analyzed. After the separation step the materials were amplified for 8 cycles using primers targeting the Tn5 adaptor. The PCR product was then purified and amplified by qPCR using primers targeting an accessible region in the human genome. **(b)** K562 scCAT-seq profiles were quality-filtered according to the number of fragments, proportion of fragments within accessible regions and detected gene numbers. **(c)** Bar plot showing the number of usable fragment at the indicated sequencing depths. **(d)** Proportion of the duplicate fragments of all K562 single cells at the sequencing depth of 400 kb. **(e)** Size distribution of chromatin accessibility fragments from an example of K562 single cell. **(f)** Percentage of the single cell chromatin accessibility fragments mapped to each nuclear chromosome and the mitochondrial genome.

(g) Correlation of chromatin accessibility between aggregate chromatin accessibility profiles and CAT-seq profile of 500 cells. **(h)** Comparison of number of usable chromatin accessibility fragments (left), proportion of fragments within the accessible regions (middle) and Pearson correlation coefficients (right) between scCAT-seq and published scATAC-seq profiles. The peaks indicated in middle panel are called based on aggregate profiles. **(i)** Correlation between aggregate gene expression profiles of all single cells and gene expression profiles generated from 500 cells. **(j)** Comparison of the number of detected genes between scCAT-seq and published scRNA-seq profiles.

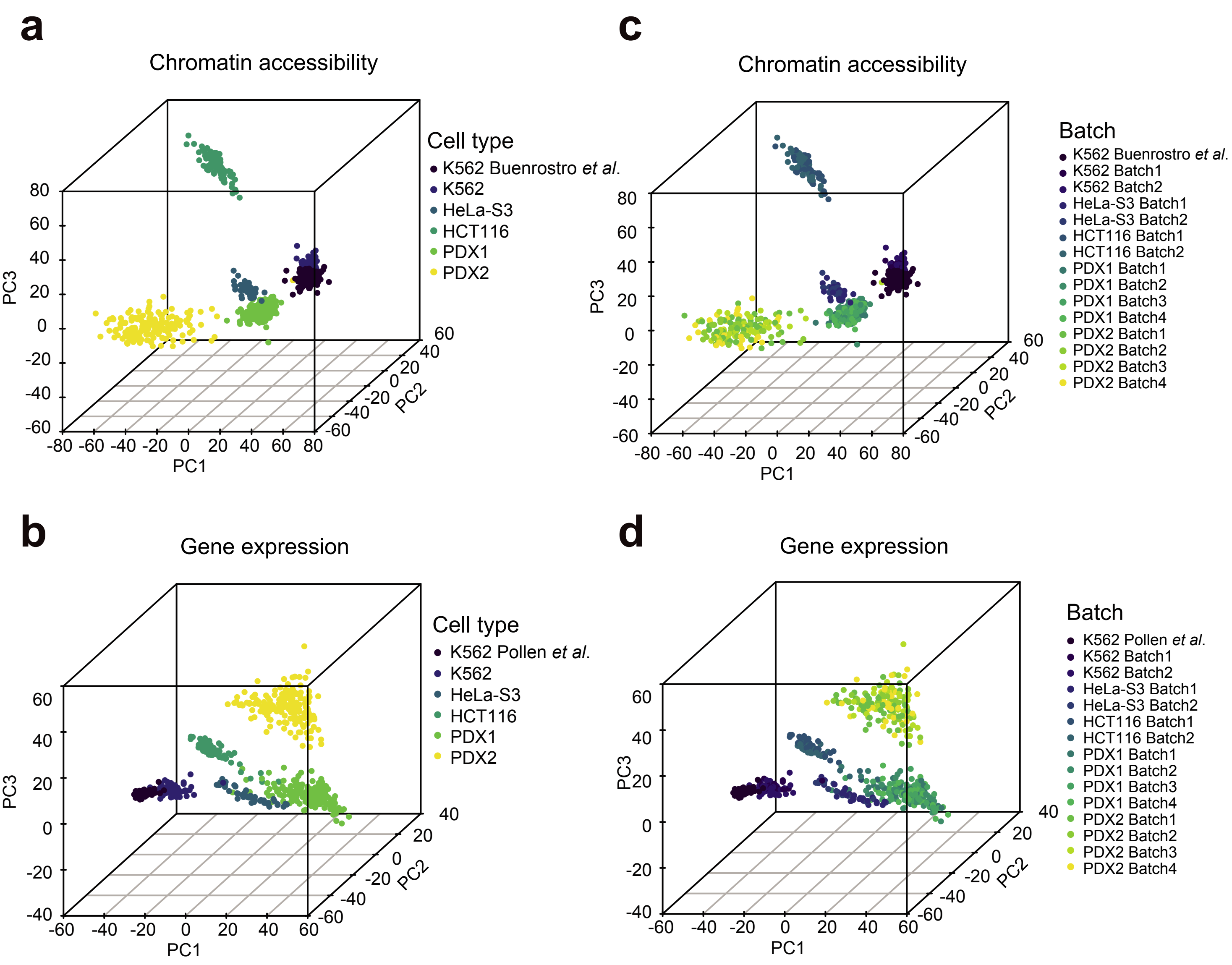

Supplementary Figure 2: Principle components analysis across diverse techniques and different batches of scCAT-seq profiles. (**a** and **c**) Principle components analysis of different batches of scCAT-seq-generated chromatin accessibility data and published datasets. (**b** and **d**) Principle components analysis of different batches of scCAT-seq-generated gene expression data and published datasets.

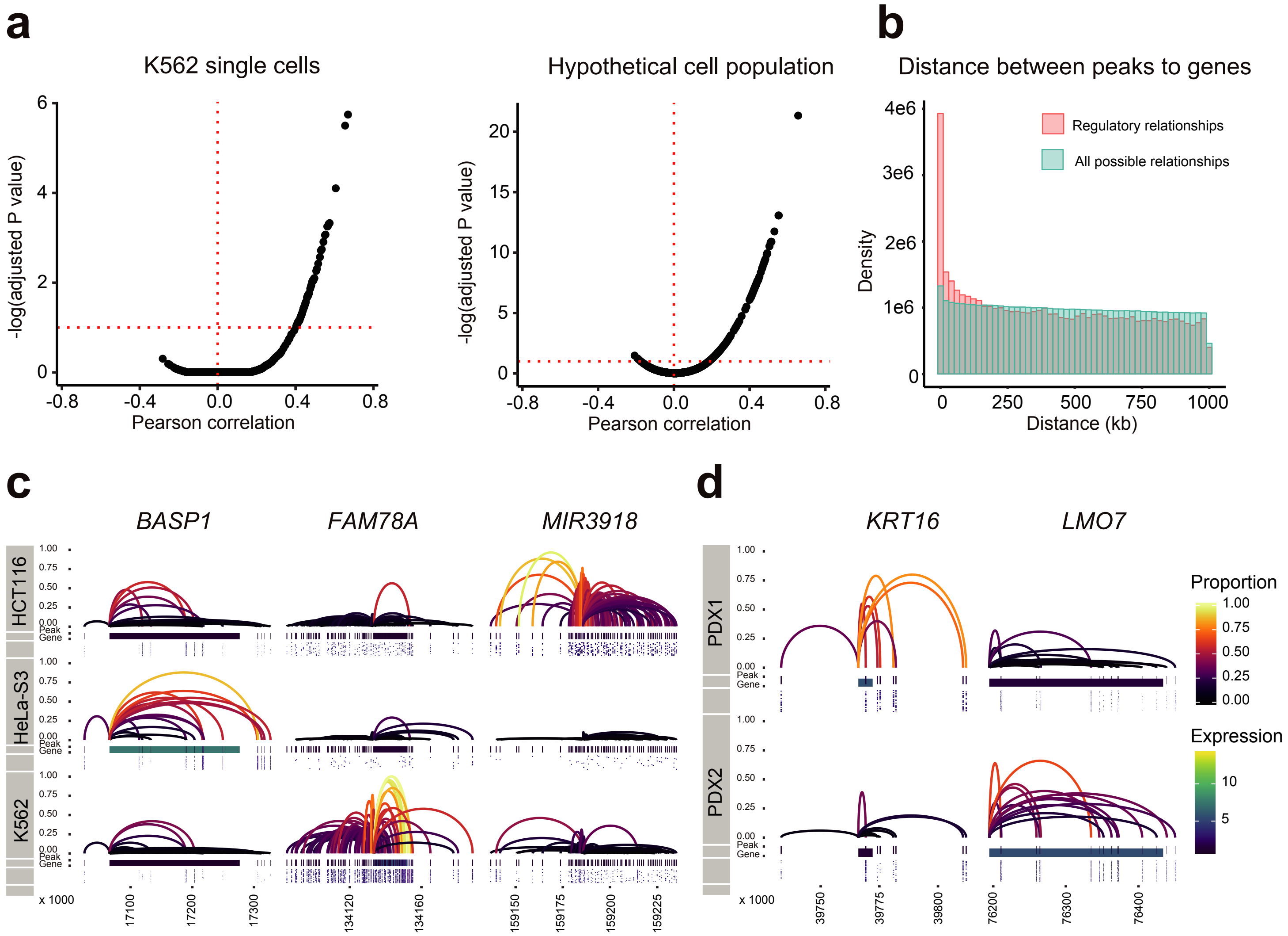

Supplementary Figure 3: scCAT-seq uncovers the regulatory relationships between CREs and genes. (a) Correlation analysis between chromatin accessibility of individual element and the putative gene expression in K562 single cells and hypothetical cell population from the three cell lines. Shown are Pearson correlation coefficients versus the Benjamini-Hochberg adjusted p-value. Significant relationships (adjusted p-value <= 0.05) are above the red dotted line. **(b)** Bar plot showing the density distribution of distances between CREs and genes in regulatory relationships (red) and random relationships (blue) **(c-d)** Regulatory relationships for the indicated genes in single cells of the three cell types **(c)** and two PDX tissues **(d)**.

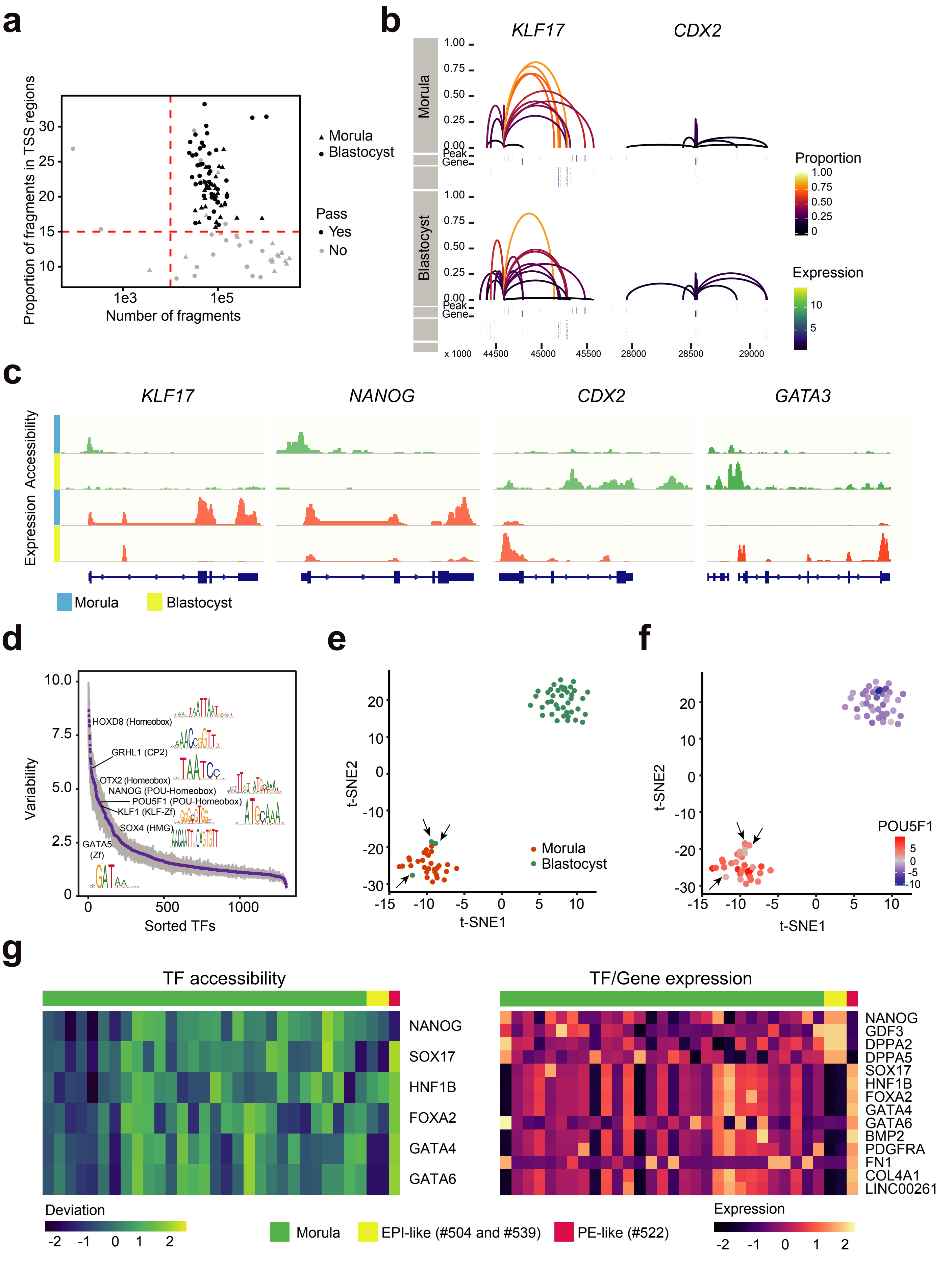

Supplementary Figure 4: Integrated profiling of chromatin accessibility and gene expression

in human pre-implantation embryos. (a) Morula and blastocyst scCAT-seq profiles were quality-filtered according to the number of fragments, proportion of fragments within promoter regions and detected gene number. **(b)** Regulatory relationships for the indicated genes in single cells of morula and blastocyst stage. **(c)** Genome browser views of chromatin accessibility and gene expression surrounding the indicated genes. **(d)** Observed cell-to-cell variability of TFs. TF families and motifs are indicated. **(e)** t-SNE plot of TF motif accessibility deviation, colored by the stage of all single cells. **(f)** t-SNE plot colored by accessibility deviation z-score of POU5F1 motif. The three blastocyst cells that are closed to the morula cells are highlighted with the black arrows. **(g)** Heatmaps showing accessibility deviation (left) and expression level (right) of the indicated TFs.
