# Supplementary material for: Deconvolution of single-cell multi-omics layers reveals regulatory heterogeneity
Source: Nat Commun. 2019 Jan 28;10:470. doi: 10.1038/s41467-018-08205-7 (PMC6349937; doi:10.1038/s41467-018-08205-7)
Supplement: Supplementary file 3 — Description of Additional Supplementary Files [file 41467_2018_8205_MOESM3_ESM.pdf]

Description of Additional Supplementary Files

File Name: Supplementary Data 1
Description: Summary sequencing statistics for all scCAT-seq profiles.

File Name: Supplementary Data 2
Description: Published datasets downloaded from ENCODE.

File Name: Supplementary Data 3
Description: Chromatin accessibility read count matrix for cell line and PDX scCAT-seq profiles used in this study.

File Name: Supplementary Data 4
Description: Gene expression read count matrix for cell line and PDX scCAT-seq profiles used in this study.

File Name: Supplementary Data 5
Description: Chromatin accessibility read count matrix for embryo scCAT-seq profiles used in this study.

File Name: Supplementary Data 6
Description: Gene expression read count matrix for embryo scCAT-seq profiles used in this study.
