# Supplementary material for: Deconvolution of single-cell multi-omics layers reveals regulatory heterogeneity
Source: Nat Commun. 2019 Jan 28;10:470. doi: 10.1038/s41467-018-08205-7 (PMC6349937; doi:10.1038/s41467-018-08205-7)
Supplement: Supplementary file 10 — Reporting Summary [file 41467_2018_8205_MOESM10_ESM.pdf]

nature research

Corresponding author(s): Roland Eils; Zhouchun Shang; Xun Xu

Reporting Summary

Nature Research wishes to improve the reproducibility of the work that we publish. This form provides structure for consistency and transparency in reporting. For further information on Nature Research policies, see Authors & Referees and the Editorial Policy Checklist.

Statistical parameters

When statistical analyses are reported, confirm that the following items are present in the relevant location (e.g. figure legend, table legend, main text, or Methods section).

n/a	Confirmed	
☒	☐	The exact sample size (*n*) for each experimental group/condition, given as a discrete number and unit of measurement
☐	☒	An indication of whether measurements were taken from distinct samples or whether the same sample was measured repeatedly
☐	☒	The statistical test(s) used AND whether they are one- or two-sided<br>*Only common tests should be described solely by name; describe more complex techniques in the Methods section.*
☐	☒	A description of all covariates tested
☐	☒	A description of any assumptions or corrections, such as tests of normality and adjustment for multiple comparisons
☐	☒	A full description of the statistics including central tendency (e.g. means) or other basic estimates (e.g. regression coefficient) AND variation (e.g. standard deviation) or associated estimates of uncertainty (e.g. confidence intervals)
☐	☒	For null hypothesis testing, the test statistic (e.g. *F*, *t*, *r*) with confidence intervals, effect sizes, degrees of freedom and *P* value noted<br>*Give P values as exact values whenever suitable.*
☒	☐	For Bayesian analysis, information on the choice of priors and Markov chain Monte Carlo settings
☐	☒	For hierarchical and complex designs, identification of the appropriate level for tests and full reporting of outcomes
☐	☒	Estimates of effect sizes (e.g. Cohen's *d*, Pearson's *r*), indicating how they were calculated
☐	☒	Clearly defined error bars<br>*State explicitly what error bars represent (e.g. SD, SE, CI)*

Our web collection on statistics for biologists may be useful.

Software and code

Policy information about availability of computer code

Data collection	No code were used to collect data.
Data analysis	For chromatin accessibility profiles, the raw reads of chromatin accessibility data were trimmed by custom's script and aligned using Bowtie. Duplicate reads were removed using Picard. MACS2 was used to call peaks. For RNA profiles, reads were mapped to hg19 genome using HISAT2. The number of read within each gene in each single cell (GENCODE, v19) were counted using GenomicAlignments package. All details were described in the Methods section.

For manuscripts utilizing custom algorithms or software that are central to the research but not yet described in published literature, software must be made available to editors/reviewers upon request. We strongly encourage code deposition in a community repository (e.g. GitHub). See the Nature Research guidelines for submitting code & software for further information.

Data

Policy information about availability of data

All manuscripts must include a data availability statement. This statement should provide the following information, where applicable:

- Accession codes, unique identifiers, or web links for publicly available datasets
- A list of figures that have associated raw data
- A description of any restrictions on data availability

All raw data have been deposited to CNGB Nucleotide Sequence Archive (accession code: CNP0000213; https://db.cngb.org/cnsa/project/CNP0000213/public/), The raw data of the cell lines have also been deposited to NCBI Sequence Read Archive (accession code: SRP167062; https://trace.ncbi.nlm.nih.gov/Traces/sra/sra.cgi?study=SRP136421). All other relevant data is available upon request.

Field-specific reporting

Please select the best fit for your research. If you are not sure, read the appropriate sections before making your selection.

- [x] Life sciences
- [ ] Behavioural & social sciences
- [ ] Ecological, evolutionary & environmental sciences

For a reference copy of the document with all sections, see nature.com/authors/policies/ReportingSummary-flat.pdf

Life sciences study design

All studies must disclose on these points even when the disclosure is negative.

Sample size	No statistical analysis was performed to predetermine sample sizes.
Data exclusions	scCAT-seq data were filtered using criteria including number of reads mapped to the genome, proportion of reads within accessible regions and the detected gene numbers (TPM > 1).
Replication	We obtained a total of 621 quality-filtered single-cell datasets comprising 42 HeLa-S3 cells, 90 HCT116 cells, 74 K562 cells, 343 PDX cells and 72 blastomeres. The single cell datasets within each group were reliably reproducible, as shown by correlation and clustering analysis in the Supplementary Figures.
Randomization	All single cells for cell lines, PDX tissues and embryos were randomly isolated.
Blinding	Blinding was not applicable to this study.

Reporting for specific materials, systems and methods

Materials & experimental systems

n/a	Involved in the study	
X		Unique biological materials
	X	Antibodies
	X	Eukaryotic cell lines
X		Palaeontology
X		Animals and other organisms
	X	Human research participants

Methods

n/a	Involved in the study	
X		ChIP-seq
X		Flow cytometry
X		MRI-based neuroimaging

Antibodies

Antibodies used

Anti-NAGOG; Abcam; ab109250; Immunostaining to authenticate human preimplantation embryos; https://www.abcam.com/nanog-antibody-epr20272-ab109250.html
Anti-SOX17; R&D; AF1924; Immunostaining to authenticate human preimplantation embryos; https://www.rndsystems.com/cn/products/human-sox17-antibody_af1924
Donkey anti-Rabbit IgG; Thermo; A21206; Immunostaining to authenticate human preimplantation embryos; https://www.thermofisher.com/antibody/product/Donkey-anti-Rabbit-IgG-H-L-Highly-Cross-Adsorbed-Secondary-Antibody-Polyclonal/A-21206
Donkey anti-Goat IgG; Thermo; A11058; Immunostaining to authenticate human preimplantation embryos; https://www.thermofisher.com/antibody/product/Donkey-anti-Goat-IgG-H-L-Cross-Adsorbed-Secondary-Antibody-Polyclonal/A-11058

Validation	All antibodies used in this research were purchased commercially. The antibodies were validated based on the information from the manufacturers' instructions.

Eukaryotic cell lines

Policy information about cell lines

Cell line source(s)	HeLa-S3 (ATCC); HCT116 (ATCC); K562(ATCC)
Authentication	STR-based method.
Mycoplasma contamination	All cell lines are tested negative for mycoplasma contamintation.
Commonly misidentified lines (See ICLAC register)	No commonly misidentified cell lines were used.

Human research participants

Policy information about studies involving human research participants

Population characteristics	All of the gametes and embryos were obtained from the donors undergoing in vitro fertilization (IVF) treatments at the Reproductive & Genetic Hospital of CITIC-XIANGYA. These donor couples, whose infertility is purely due to tubal factors and the female patients is under ages of 35 years old.
Recruitment	Reproductive & Genetic Hospital of CITIC-XIANGYA was in charge of recruiting research donors in this study. Before giving consent, volunteers were given information about the research project. All human gametes were collected after receiving written informed consent from the donors. The recruitment was through word-of-mouth. No potential self-selection bias or other biases were present in this study.
